# Amplitude Dispersion Compensation for Damage Detection Using Ultrasonic Guided Waves

**DOI:** 10.3390/s16101623

**Published:** 2016-09-30

**Authors:** Liang Zeng, Jing Lin, Liping Huang, Ming Zhao

**Affiliations:** 1 School of Mechanical Engineering, Xi’an Jiaotong University, Xi’an 710049, China; liangzeng@mail.xjtu.edu.cn (L.Z.); lipinghuang@stu.xjtu.edu.cn (L.H.); zhaomingxjtu@mail.xjtu.edu.cn (M.Z.); 2 State Key Laboratory for Manufacturing Systems Engineering, Xi’an Jiaotong University, Xi’an 710049, China

**Keywords:** guided wave, dispersion, time reversibility, testing resolution

## Abstract

Besides the phase and group velocities, the amplitude of guided wave mode is also frequency dependent. This amplitude dispersion also influences the performance of guided wave methods in nondestructive evaluation (NDE) and structural health monitoring (SHM). In this paper, the effects of amplitude dispersion to the spectrum and waveform of a propagating wave-packet are investigated. It is shown that the amplitude dispersion results in distortion in the spectrum of guided wave response, and thus influences the waveform of the wave-packet. To remove these effects, an amplitude dispersion compensation method is established on the basis of Vold–Kalman filter and Taylor series expansion. The performance of that method is then investigated by experimental examples. The results show that with the application of the amplitude dispersion compensation, the time reversibility could be preserved, which ensures the applicability of the time reversal method for damage detection. Besides, through amplitude dispersion compensation, the testing resolution of guided waves could be improved, so that the structural features located in the close proximity may be separately identified.

## 1. Introduction

In general, in-situ structural health monitoring system requires the capability of inspecting a relatively large area and providing reliable diagnosis (e.g., defect type, location and severity) instantaneously. With the advantages of fast scanning capabilities, low cost, long-range inspection, and testing inaccessible or complex components, guided waves have attracted considerable attentions in structural health monitoring (SHM) [1].

Small and conformal transducers, e.g., piezoceramics transducers (PZTs) and inter digital transducers (IDTs), either being surface mounted or embedded leave-in-place on the structure, have been widely studied for generating and receiving guided waves for structural health monitoring. The IDTs can activate single mode waves with controllable wavelength and even dispersion properties that propagate in the desired direction [2,3,4]. Besides, they are flexible and can be easily shaped to cope with curved structural surfaces [5,6]. However, they exhibit a relatively weak driving power and, therefore, some related inconveniences may appear limiting the area of practical applications [1,7]. The PZTs are the most widely used transducers in the development of SHM systems because of the advantages including superior electro-mechanical coupling, wide frequency responses, easy integration and activation, low power consumption and low cost [8,9]. However, PZTs-generated guided waves are unavoidably dispersive and contain multiple wave modes, which makes the guided wave signals are hard to be interpreted. Accordingly, considerable studies have been conducted into understanding the basic principles (i.e., dispersive and multi-modal characteristics) and applications of guided waves [10,11,12,13,14,15,16,17,18,19].

Actually, associated with the guided wave transmitting, propagating and sensing processes, the guided wave components at different frequency values are non-uniformly scaled. Giurgiutiu [20] investigated the variation of displacement of rectangular piezoelectric wafer active sensors (PWAS) with frequency for each guided wave mode. Raghavan and Cesnik [21] modeled the displacement response of a circular piezoelectric actuator in isotropic plates on the basis of the 3-D linear elasticity equations. Park and Sohn [22] analyzed the amplitude response of guided wave mode via Mindlin plate theory. According to these studies, the amplitude of guided wave mode is frequency dependent, which is associated with the dimensions of PZT and the fundamental properties of the plate (e.g., density, flexural stiffness and thickness). Besides, the attenuation as guided wave mode propagates in the structure also results in frequency dependency in its amplitude. Neau [23] extended the analytical model of guided wave to take into account the viscosity of the insonified material, and calculated the wave attenuation caused by the material absorption. Schubert and Herrmann [24] investigated the frequency dependent material damping for guided wave propagating in viscoelastic composites. These studies have already revealed the factors that result in frequency dependency in the amplitude of guided wave mode. However, there is still a lack of systematic analysis for the influence of this amplitude dispersion on guided wave based damage detection.

In this paper, the effects of the amplitude dispersion to a propagating wave-packet are investigated systematically. In addition, an amplitude dispersion compensation method is proposed to remove these effects and improve the performance of guided wave methods for damage detection. The rest of this paper is organized as follows. In Section 2, the theories associated with the amplitude dispersion have been reviewed. Subsequently, the influences of amplitude dispersion to the spectrum and the waveform of guided wave mode are investigated in Section 3. In Section 4, an amplitude dispersion compensation method is proposed. The specimen and experimental setup are then introduced in Section 5. In Section 6, the effects of the amplitude dispersion to guided wave based damage detection, and the effectiveness of the proposed amplitude dispersion compensation method are investigated. Finally, conclusions are drawn in Section 7.

## 2. Definition of Amplitude Dispersion

Considering that two guided wave transducers are positioned on a plate (or layer) serving as the transmitter and receiver, respectively, the entire system comprising the instrumentation, transducers and structures may be well-modeled as a linear system [25]. Hence, the response of a particular guided wave mode at the location of the receiver could be expressed in the general form:

(1) $$u\left(x,t\right)=\int_{-\infty }^{\infty }H\left(x,\omega \right)F\left(\omega \right)e^{-j\omega t}d\omega ,$$

where *F*(*ω*) is the Fourier transform of the input signal *f*(*t*), and *H*(*x*,*ω*) is the system transfer function of that mode, i.e.,

(2) $$H\left(x,\omega \right)=\left|H\left(x,\omega \right)\right|e^{j\xi \left(\omega \right)x},$$

where *ξ*(*ω*) is the (angular) wavenumber as a function of (angular) frequency, which is related to the phase velocity *c_p_* by the equation *ξ* = *ω*/*c_p_*. The term *e^jξ^*^(*ω*)*x*^ is associated with the velocity dispersion characteristics of guided wave mode. It means that the energy in a wave-packet propagates at different speeds depending on the frequency.

Actually, the amplitude spectrum of the transfer function, |*H*(*x*,*ω*)|, also varies with respect to the frequency, which is associated with the transmitting, propagating and sensing processes.

### 2.1. Transmitting

If PWAS is used as an actuator, the exited amplitude of guided wave modes at a certain frequency depends on the actuator itself. Assuming ideal bonding between the PWAS (rectangular, of length *l_a_* = 2*a*) and the plate (of thickness *d* = 2*h*), the displacement wave solution could be obtained as [20],

(3) $${u_{x}\left(x,t\right)|}_{y=h}=-\frac{a\tau_{0}}{\mu }e^{-i\omega t}\left[\sum_{\xi^{S}}\frac{\sin\left(\xi^{S}a\right)}{\xi^{S}}\frac{N_{S}\left(\xi^{S}\right)}{D_{S}^{\prime }\left(\xi^{S}\right)}e^{i\xi^{S}x}+\sum_{\xi^{A}}\frac{\sin\left(\xi^{A}a\right)}{\xi^{A}}\frac{N_{A}\left(\xi^{A}\right)}{D_{A}^{\prime }\left(\xi^{A}\right)}e^{i\xi^{A}x}\right],$$

where the PWAS is modeled as causing surface shear stress *τ* = *τ*_0_(*x*)*e^iωt^* along the piezo edges, *λ* and *μ* are the Lame constants for the plate material (ideally elastic, isotropic and homogeneous), *ξ* is the wavenumber of guided wave mode, and

(4) $$\begin{array}{l} p^{2}=\frac{\omega^{2}}{c_{L}^{2}}-\xi^{2},\;q^{2}=\frac{\omega^{2}}{c_{T}^{2}}-\xi^{2},\;c_{L}=\sqrt{\frac{\lambda +2\mu }{\rho }},\;c_{T}=\sqrt{\frac{\mu }{\rho }}\\ N_{S}=\xi q\left(\xi^{2}+q^{2}\right)\cos pd\cos qd\\ D_{S}={\left(\xi^{2}-q^{2}\right)}^{2}\cos pd\sin qd+4\xi^{2}pq\sin pd\cos qd\\ N_{A}=\xi q\left(\xi^{2}+q^{2}\right)\sin pd\sin qd\\ D_{A}={\left(\xi^{2}-q^{2}\right)}^{2}\sin pd\cos qd+4\xi^{2}pq\cos pd\sin qd \end{array},$$

where *ρ* is the material density.

If the PWAS spans half the wavelength multiplied with odd integers, this results in a maximum output signal. If the PWAS spans areas containing both positive and negative strains, the amplitude is reduced. This results in different response amplitudes of a guided wave mode at different frequencies, as it propagates with different speeds at different frequencies and the wavenumber *ξ* is related to the phase speed *c_p_* by the equation *ξ* = *ω*/*c_p_*.

### 2.2. Propagating

The attenuation as guided wave mode propagates in the plate depends on many different factors. The most important ones are geometric spreading, material damping, dissipation into adjacent media and wave attenuation due to velocity dispersion [24].

(a) Geometric spreading is related to amplitude loss due to the growing length of a wave front departing into all directions from a source. It causes the amplitude of a wave-packet to decrease in proportion to the square root of the increase in its wave front,
  (5) $$A_{2}=A_{1}\cdot \sqrt{d_{1}/d_{2}},$$
  where *A*_1_ and *A*_2_ denote the wave amplitudes, and *d*_1_ and *d*_2_ are the distances from the source. This type of attenuation is independent of the frequency.
(b) The material damping describes the energy dissipation of a wave-packet due to non-perfect elastic material behavior. In composite structures, it can be expressed as follows,
  (6) $$A_{2}=A_{1}e^{-k^{\prime \prime }\left(d_{2}-d_{1}\right)},$$
  where *k*″ is the attenuation factor. It is a function of the frequency, and can be measured experimentally or predicted using different material models [26]. This material damping introduces frequency dependency in the amplitude of a propagating guided wave mode.
(c) Dissipation into adjacent media (i.e., leakage) describes how much energy is lost into adjacent media. If the adjacent media is air, the attenuation could be ignored [24].
(d) The spreading of a wave-packet in time due to velocity dispersion reduces its absolute amplitude. However, this energy spreading depends on the steepness of the velocity dispersion curves, and does not influence the amplitude spectra of guided wave signals (see Equation (2)). In addition, if the velocity dispersion is compensated, the energy spreading in time can be gathered again because of energy conservation.

In summary, the second factor, material damping, dominates the frequency dependency of wave attenuation.

### 2.3. Sensing

The ratio of displacement of a particular mode to applied force when both quantities are measured at the same location and direction in the cross section is defined as the excitability of that mode. It is a function of frequency. If the receiving and transmitting transducers were exactly the same, the detectability and excitability would have the same value [27].

This study is mainly concerned with the amplitude dispersion, which is independent of the velocity dispersion. Therefore, only the frequency dependency in the amplitude spectrum of the system transfer function, |*H*(*x*,*ω*)|, is considered. In this case, the amplitude dispersion is defined as:

(7) G(ω)=|H(x,ω)|=E(ω)A(x,ω)D(ω),

where *E*(*ω*), *A*(*x*,*ω*) and *D*(*ω*) are the excitability, attenuation and detectability of the guided wave mode at the transmitting, propagating and sensing processes, respectively.

## 3. Effects of Amplitude Dispersion

**Figure 1:**
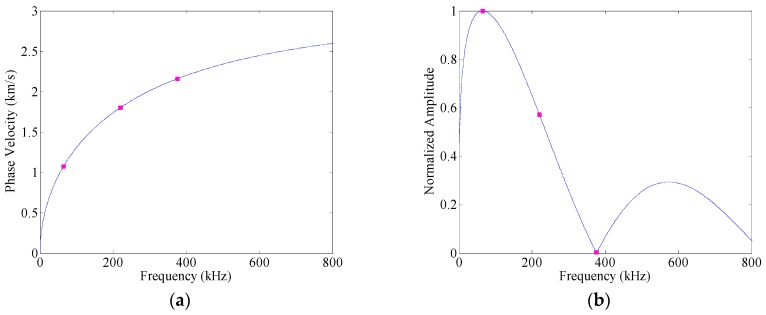
Phase velocity dispersion curve (**a**); and excitability (**b**) of the A0 Lamb wave mode.

To illustrate the effects of the amplitude dispersion, the case how guided wave-packet excited by a Hanning windowed toneburst as the A0 Lamb wave mode propagates in a 2-mm thick 2024-T3 aluminum plate (i.e., density: 2780 kg/m^3^, Young’s modulus: 73.1 GPa, Poisson’s ratio: 0.33) is modeled. The A0 Lamb wave mode is excited by a rectangular PWAS with the dimensions of 7 mm (length) × 7 mm (width) × 0.2 mm (thickness), the compliance of 1.53 × 10^−11^ m^2^/N, and the d_31_ coefficient of −1.75 × 10^−10^ m/V. The phase velocity dispersion curves of A0 Lamb wave mode is calculated from the Rayleigh–Lamb equations and is shown in Figure 1a.

In the first case, it is assumed that the amplitude of system transfer function of A0 Lamb wave mode remains constant over the excitation frequency band. Thus, the received time-trace is predicted as,

(8) $$u\left(x,t\right)=\int F\left(\omega \right)e^{j\left(\xi \left(\omega \right)x-j\omega t\right)}d\omega ,$$

where *F*(*ω*) is the Fourier transform of the toneburst excitation signal *f*(*ω*). In this case, only the velocity dispersion alters the waveform of the propagating A0 mode.

**Figure 2:**
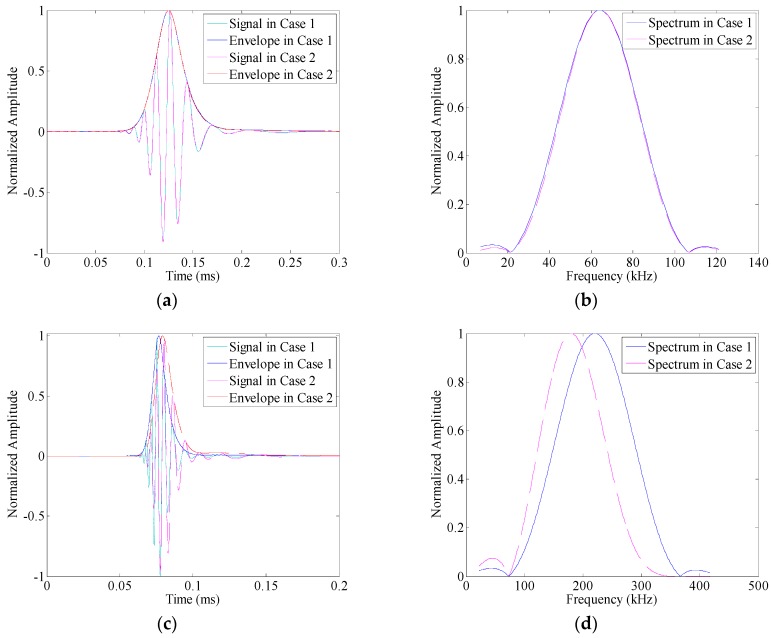

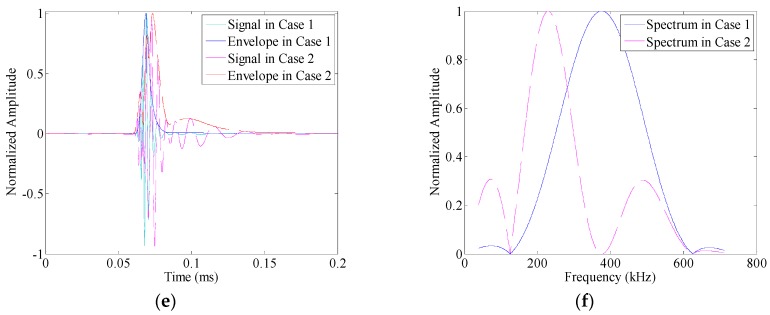
The A0 mode responses and the amplitude spectra evolution in Cases 1 and 2 as the center frequency of the toneburst excitation varies: (**a**,**b**) 64 kHz; (**c**,**d**) 220 kHz; and (**e**,**f**) 375 kHz.

Three-cycle Hanning windowed toneburst signals with center frequencies of 64 kHz, 220 kHz and 375 kHz are applied to the transmitter as the input signals. The propagation distance *x* is 200 mm. The received time-traces calculated from Equation (8) are shown in Figure 2a,c,e, respectively. It can be seen that an element of the A0 wave package at a relatively lower frequency arrives later than the one at a higher frequency, which is associated with the group velocity dispersion. Besides, Figure 2b,d,f show the spectra of these A0 Lamb wave responses (the blue solid lines). From Equation (8), it can be observed that the dispersive term, exp(*jξ*(*ω*)*x*), controls the phase of Lamb wave response, but does not influence its amplitude spectrum. Hence, these spectra exactly match the spectra of the associated toneburst excitations.

Actually, the amplitude of the system transfer function is frequency dependent, which is associated with the dimensions of PWAS and the fundamental properties of the plate. As mentioned in Section 2, if a toneburst signal *f*(*ω*) is applied to the transmitter, the A0 mode Lamb wave response satisfies,

(9) $$u\left(x,t\right)=\;\int G\left(\omega \right)F\left(\omega \right)e^{j\left(\xi \left(\omega \right)x-j\omega t\right)}d\omega ,$$

In metallic plates, the material damping due to the viscoelasticity of the material does not influence the amplitude–frequency dependency of system transfer function significantly [18]. In addition, since the receiving and transmitting PWAS are of the same geometrical and physical properties, the excitability and the detectability would have the same value.

Accordingly, in the second case, the received time-trace is predicted as,

(10) $$u\left(x,t\right)=\int E{\left(\omega \right)}^{2}F\left(\omega \right)e^{j\left(\xi \left(\omega \right)x-j\omega t\right)}d\omega ,$$

Equation (3) gives the relation between the displacement of a particular mode and the applied force. Hence, the excitability *E*(*ω*) of A0 mode is calculated from that equation and shown in Figure 1b. It is a non-negative real function of angular frequency *ω*, and thus only influences the amplitude spectrum of guided wave response. Especially, the amplitude spectrum of guided wave mode at each frequency point is the product of the amplitude dispersion of that mode and the spectrum of the excitation, i.e.,

(11) $$\left|U\left(x,\omega \right)\right|=E{\left(\omega \right)}^{2}\left|F\left(\omega \right)\right|,$$

The amplitude dispersion would cause the spectra of wave-packets propagating in the structure to be distorted compared to the spectrum of the input signal. To measure its effect, an influence index is defined as,

(12) $$Influence\;Index=1-\frac{\sum_{k=1}^{K}\left(X_{k}-\frac{1}{K}\sum_{k=1}^{K}X_{k}\right)\left(Y_{k}-\frac{1}{K}\sum_{k=1}^{K}Y_{k}\right)}{\sqrt{\sum_{k=1}^{K}{\left(X_{k}-\frac{1}{K}\sum_{i=1}^{K}X_{k}\right)}^{2}}\sqrt{\sum_{k=1}^{K}{\left(Y_{k}-\frac{1}{K}\sum_{i=1}^{K}Y_{k}\right)}^{2}}},$$

where *K* is the length of the data set, *X* is the spectrum of the input signal and *Y* is the spectrum of the guided wave response (Equation (11)).

The input signals are three-cycle Hanning windowed toneburst signals centered at 64 kHz, 220 kHz and 375 kHz, respectively. For comparison, the responses calculated from Equation (10) are also displayed in Figure 2a,c,e, and their spectra are shown in Figure 2b,d,f, respectively.

A threshold (i.e., 0.1) is set for bandwidth definition. When the center frequency of the toneburst excitation takes 64 kHz, the frequency bandwidth of excitation could be estimated as [29, 99] kHz. As shown in Figure 1b, the excitability of A0 mode varies with the frequency at this range, and its peak sits at the central frequency of the excitation (i.e., 64 kHz). The amplitude dispersion does not influence the spectrum of A0 mode significantly (Figure 2b). The influence index is calculated as 1.71 × 10^−4^, indicating the similarity between the spectrum of A0 mode response and that of the input signal. Accordingly, the received time traces in Cases 1 and 2 coincide with each other well (Figure 2a). When the central frequency of the toneburst excitation takes 220 kHz, the influence index increases to 0.219. Figure 2d shows the spectrum of A0 mode response in Case 2 as the purple dashed-line. The peak frequency is 179 kHz, much smaller than that of the excitation. Besides, the frequency bandwidth of the response is [87, 292] kHz, while that of the excitation is [98, 341] kHz. These distortions in spectrum due to amplitude dispersion results in the deviation of the waveform of A0 mode response, as shown in Figure 2c. Hilbert transfer is then applied to calculate the envelopes of the received time traces, and a threshold (i.e., 0.1) is set on these envelopes to define the time duration of wave packet. Without amplitude dispersion (i.e., Case 1), the peak of envelope sits at 0.0767 ms, and the time range of wave packet is [0.0675, 0.0909] ms. In comparison, due to the effects of amplitude dispersion (i.e., Case 2), the peak of envelope moves to 0.0793 ms, and the time range of wave packet changes to [0.0693, 0.0959] ms. As the central frequency further increases to 375 kHz, the influence index is calculated as 0.9439, indicating the significant distortion of the spectrum (Figure 2f). As shown in Figure 2e, the waveform of the A0 mode response changes obviously due to amplitude dispersion. Especially, there are four local maxima at the envelope of A0 mode response in Case 2. In this case, it is hard to identify the actual number of wave packets, and may lead to a false alarm.

**Figure 3:**
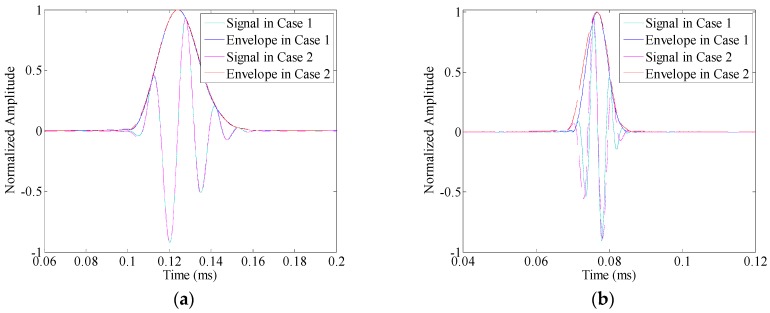

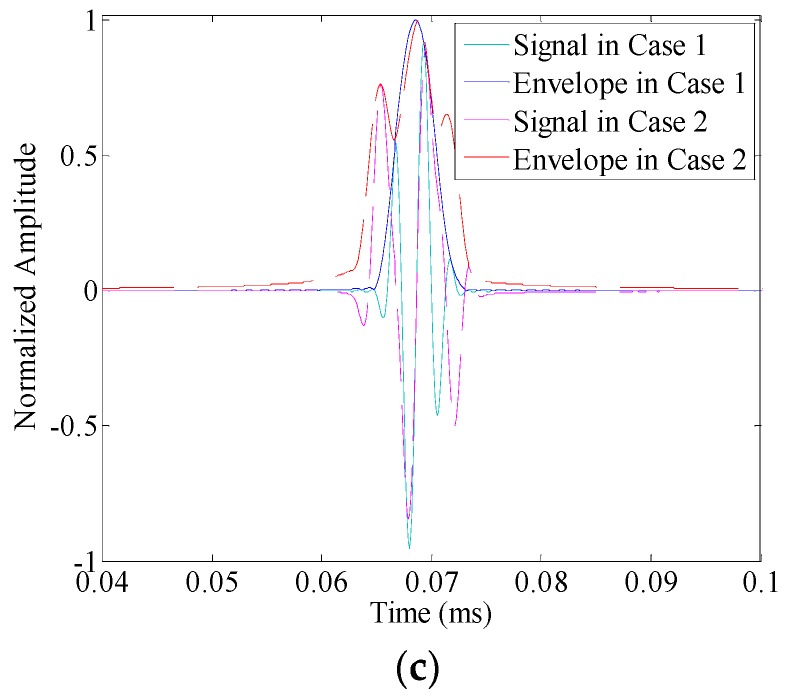
The A0 mode waveforms after dispersion compensation: (**a**) 64 kHz; (**b**) 220 kHz; and (**c**) 375 kHz.

For further illustration, the linear mapping technique in [28] is applied to the A0 mode responses to compensate the velocity dispersion effects, and thus highlight the influences of amplitude dispersion. The associated results are shown in Figure 3a–c, respectively. Without amplitude dispersion (i.e., Case 1), the A0 mode responses could be restored to the exact shape of the original input signal. However, in Case 2, the amplitude dispersion still influences the waveform of the results. Especially when the central frequency of the toneburst excitation takes 220 kHz or 375 kHz, the waveform deviations caused by the amplitude dispersion could be obviously observed in Figure 3b,c, respectively. These deviations, wave-packet duration increasing and envelope fluctuation (multiple local maxima), are undesirable in guided wave testing. The former one reduces the testing resolution that can be achieved, and the latter one increases the complexity of signal interpretation and the risk of error alarm.

## 4. Compensation of Amplitude Dispersion

The effect of amplitude dispersion is that the wave components at different frequency values are non-uniformly scaled. It results in the deviation of the waveform, and thus influences the identification of damage. To compensate its effects, a two-step amplitude dispersion compensation strategy is established. In the following section, the two main steps, i.e., amplitude dispersion estimation and amplitude dispersion compensation, will be introduced.

### 4.1. Amplitude Dispersion Estimation

In practical applications, there are multiple guided wave modes that can propagate in the structure. All these modes are dispersive and quite possible to overlap with each other, yielding the problem of mode separation. Vold–Kalman filter is a powerful time-varying filter [29,30]. Its center frequency and bandwidth can be adjusted dynamically according to the signal’s time–frequency distribution. Besides, it can extract all concerned components simultaneously without introducing any phase bias into the extracted waveforms [31]. Hence, it is particularly suitable for mode extraction in guided wave signals.

VKF is performed based on the data equation and structural equation. Data equation represents the measured signal as a summation of interested components, non-concerned components and noise, which is given by the following equation,

(13) $$y\left(n\right)=\sum_{k}x_{k}\left(n\right)+\eta \left(n\right),$$

where *y*(*n*) is the measured signal, *k* = 1,…, *K* represents the components to be extracted; and *η*(*n*) represents not-concerned components and the noise.

It is well acknowledged that every real-valued *x_k_*(*n*) can be expressed in its equivalent analytical form. As a result, the data equation can also be written as

(14) $$y\left(n\right)=\sum_{k=\pm 1,\cdots ,\pm K}a_{k}\left(n\right)e^{j\theta_{k}\left(n\right)}+\eta \left(n\right),$$

where *a_k_*(*n*) is the complex envelope of *x_k_*(*n*), and *a*_−*k*_(*n*) is the complex conjugate of *a_k_*(*n*), thus *x_k_*(*n*) is real. *θ_k_*(*n*) is the instantaneous phase which is determined in Equation (15).

(15) $$\theta_{k}\left(n\right)=\sum_{m=0}^{n}\omega_{k}\left(\omega \right)\Delta T,$$

where *ω_k_*(*m*) is the instantaneous angular frequency of *x_k_*(*m*) at the *m*th sampling instant.

During the propagation in the structure, the envelope of each mode *a_k_*(*n*) changes smoothly. Hence, it could be approximated by a low-order polynomial function (usually the order *s* ≤ 3). This could be mathematically expressed by the structural equation as,

(16) $$\nabla^{s}a_{k}\left(n\right)=\varepsilon_{k}\left(n\right),$$

where $\nabla^{s}$ denotes the difference operator of order *s*, *ε_k_*(*n*) is an unknown high order error term in *a_k_*(*n*).

The structural equation and data equation of VKF can be expressed by the matrix form as,

(17) $$Aa_{k}=\varepsilon_{k},$$

(18) $$y-\sum_{k=1}^{K}C_{k}a_{k}=\eta ,$$

where

(19a) $$a_{k}={\left[a_{k}(1),\cdots ,a_{k}(N)\right]}^{\tau };\;\varepsilon_{k}={\left[\varepsilon_{k}(1),\cdots ,\varepsilon_{k}(N)\right]}^{\tau },$$

(19b) $$y={\left[y(1),\cdots ,y(N)\right]}^{\tau };\;C_{k}=diag\left\{e^{j\theta_{k}(1)},\cdots ,e^{j\theta_{k}(N)}\right\};\;\eta ={\left[\eta (1),\cdots ,\eta (N)\right]}^{\tau },$$

where the superscript *τ* denotes the transpose operator. **A** is an *N* × *N* matrix determined by the difference order*s*. For instance, if *s* takes 2, it satisfies,

(19c) $$A=\left[\begin{array}{cccccccc} -2 & 1 & 0 & 0 & 0 & \cdots & 0 & 0\\ 1 & -2 & 1 & 0 & 0 & \cdots & 0 & 0\\ 0 & 1 & -2 & 1 & 0 & \cdots & 0 & 0\\ \vdots & \vdots & \vdots & \vdots & \vdots & \cdots & \vdots & \vdots \\ 0 & 0 & 0 & 0 & 0 & 0 & 1 & -2 \end{array}\right],$$

To solve structural Equation (17) and data Equation (18), a loss function of VKF is constructed as follows,

(20) $$J=\sum_{k=1}^{K}\varepsilon_{k}^{H}R_{k}^{\tau }R_{k}\varepsilon_{k}+\eta^{H}\eta ,$$

Here, superscript *H* denotes the Hermitian transpose; and **R***_k_* = *diag*{*r_k_*(1),…,*r_k_*(*N*)} is the weighting factor matrix which determines the instantaneous bandwidth in the filtering process. If the difference order *s* takes 2, the relationship between weighting factor *r_k_*(*i*) and instantaneous bandwidth Δ*f_i_* is given by Equation (21).

(21) $$r_{k}\left(i\right)=\sqrt{\frac{\sqrt{2}-1}{6-8\cos\left(\pi \Delta f_{i}\right)+2\cos\left(2\pi \Delta f\right)}},$$

By minimizing the loss function, Equation (20) is subject to

(22) $$\frac{dJ}{da_{i}^{H}}=B_{i}a_{i}+C_{i}^{H}\sum_{k=1,k\neq i}^{K}C_{k}a_{k}-Y_{i}=0,$$

where $B_{i}=A^{\tau }R_{i}^{\tau }R_{i}A+E$, **E** is the unity matrix; $Y_{i}=C_{i}^{H}y$.

The global solution for **a***_k_* in Equation (22) can be obtained using preconditioned conjugate gradient (PCG) algorithm. Tuma [30] and Guo [32] present the details about the numerical implementation of that method.

As mentioned, each mode in guided wave signals can be represented by its analytical form as *a_k_*(*n*)exp(*jθ_k_*(*n*)). The envelope *a_k_*(*n*) can be obtained via VKF from Equation (17), and the carrier wave exp(*jθ_k_*(*n*)) is determined according to Equation (18). On this basis, the waveform of each mode could be obtained as the product of the envelope and its carrier wave. On this basis, the transfer function of each mode could be estimated from the relation between the input and the extracted response associated with that mode. Finally, the amplitude dispersion curve could be calculated as the magnitude spectrum of the transfer function of that mode.

### 4.2. Amplitude Dispersion Compensation

From Equation (9), it can be seen that the amplitude dispersion places an amplitude modulation on the guided wave signals. A direct way to compensate its effects could be achieved by an inverse filter procedure, i.e.,

(23) $$\tilde{u}\left(x,t\right)=\int \frac{U\left(x,\omega \right)}{\tilde{G}\left(\omega \right)}e^{j\omega t}d\omega =\int \frac{G\left(\omega \right)}{\tilde{G}\left(\omega \right)}F\left(\omega \right)e^{j\left(\xi \left(\omega \right)x-j\omega t\right)}d\omega ,$$

Here, *ũ*(*x*,*t*) is the result after amplitude modification, *U*(*x*,*ω*) is the Fourier transform of the guided wave signal *u*(*x*,*t*), and $\tilde{G}\left(\omega \right)$ is the estimated amplitude dispersion curve. However, this inverse filter procedure faces a big challenge, i.e., it is in stable and noise may be amplified, which means it cannot be employed for practical applications. To overcome this problem, a modified strategy is proposed as follows.

First, the estimated amplitude dispersion curve is normalized by its maximum value at the excitation frequency range, so that $\tilde{G}\left(\omega \right)$ belongs to the range of [0, 1].

For simplicity, let $y=\tilde{G}\left(\omega \right)$. Subsequently, a Taylor series expansion is introduced to replace the inverse filter as,

(24) $$\frac{1}{y}=1-\left(y-1\right)+{\left(y-1\right)}^{2}-{\left(y-1\right)}^{3}+\cdots \;=\sum_{n=0}^{\infty }{\left(1-y\right)}^{n},$$

In practice, it is impossible to calculate the value of Equation (24) when the polynomial order is infinite. Hence, the Taylor series expansion needs to be cut off as,

(25) $$\frac{1}{y}\approx \sum_{n=0}^{N}{\left(1-y\right)}^{n}=\sum_{n=0}^{N}{\left(1-\tilde{G}\left(\omega \right)\right)}^{n},$$

Accordingly, the amplitude compensation could be achieved as,

(26) $$\tilde{u}\left(x,t\right)\approx \frac{1}{2\pi }\int_{-\infty }^{\infty }U\left(x,\omega \right)\left[\sum_{n=0}^{N}{\left(1-\tilde{G}\left(\omega \right)\right)}^{n}\right]e^{j\omega t}d\omega ,$$

From this equation, it can be concluded that the accuracy of the amplitude dispersion compensation is determined by how accurately the estimated amplitude dispersion curve represents the actual characteristic that guided wave signal has been subjected to. In addition, the order of the Taylor polynomial *N* is a crucial parameter needed to be chosen properly. It provides a compromise between the compensation degree and the resistance to noise. A high order could enhance the compensation degree of the amplitude dispersion. However, the result would be more sensitive to the noises included in the raw signal *u*(*x*,*t*) and the error in the estimated amplitude dispersion curve. In contrast, a low order could improve its robustness in noisy conditions; however, the amplitude dispersion cannot be fully compensated.

## 5. Experimental Setup

**Figure 4:**
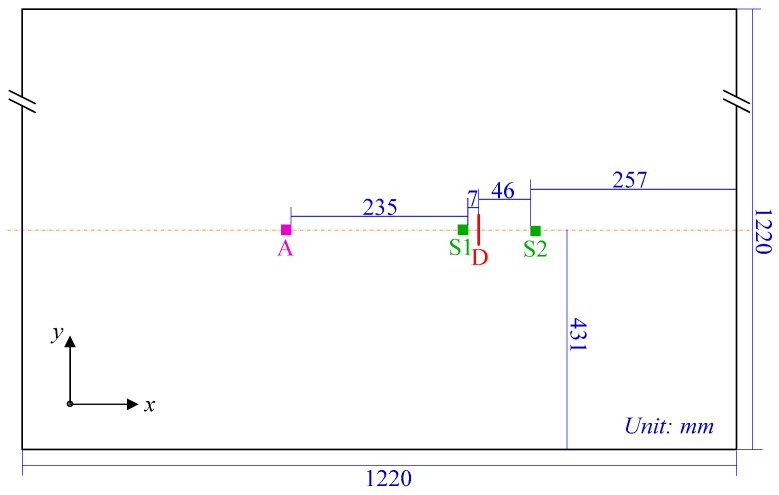
Schematic diagram of the aluminum plate and the configuration of PWAS transducers.

The experimental setup consists of an Agilent 33120A waveform generator and a Tektronix TDS5034B digital phosphor oscilloscope. The waveform generator is used to generate a linear chirp with its frequency sweeps from 1 MHz down to 5 kHz over a Hanning window with the duration of 500 μs. The Tektronix TDS5034B digital phosphor oscilloscope is used for data acquisition of the response signals. The specimen is a 2024-T3 aluminum plate with the dimensions of 1220 mm × 1220 mm × 1 mm. A simulated crack (marked as D in Figure 4) that is 19 mm in length and 0.127 mm in width is manufactured in the specimen. Figure 4 shows the schematic diagram of the specimen and the configuration of the PWAS transducers (rectangular PZT, APC850, with dimensions of 7 mm × 7 mm × 0.3 mm). PWAS A serves as the actuator to generate guided waves, and PWAS S1 and S2 serve as the sensors to record guided waves propagating in the structures.

**Figure 5:**
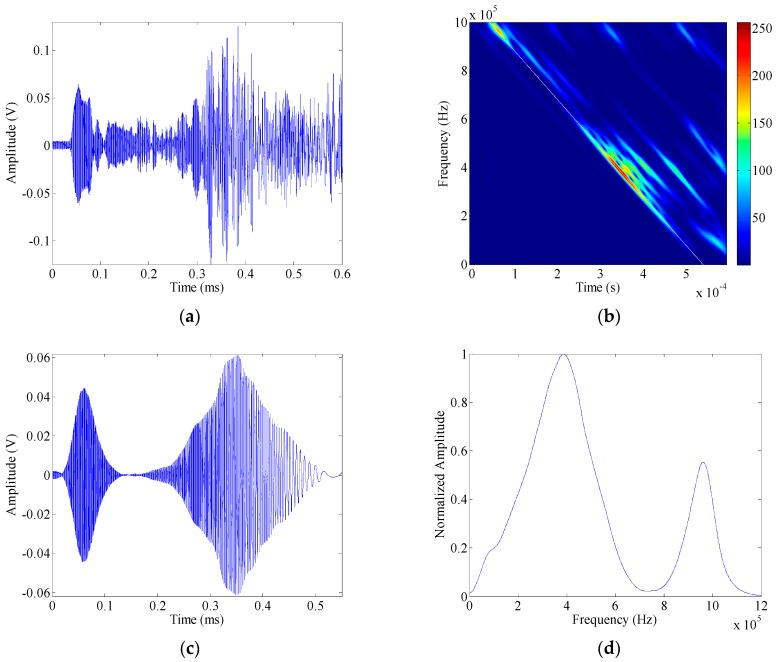
Guided wave response captured by the sensor PWAS S1 (**a**); its CT spectrogram (**b**); S0 mode extracted from the raw guided wave signal (**c**); and the amplitude dispersion curve of S0 mode (**d**).

Figure 5a shows the guided wave signal captured by the sensor (PWAS S1). The chirplet transform (CT) is then employed to investigate the dispersion effects of the guided wave modes, which is defined as,

(27) $$C_{x}\left(t,f,c\right)=\int_{-\infty }^{\infty }x\left(t+\tau \right)\omega_{T}\left(\tau \right)e^{-j2\pi \frac{c}{2}\tau^{2}}e^{-j2\pi f\tau }d\tau ,$$

where *x*(*t*) is the associated response, *t* and *f* are the time and frequency indices, respectively; and *ω_T_*(*τ*) is the window function of length *T*, and centered at time *τ* = 0. The parameter *c* takes the chirp rate of linear chirp excitation. Figure 5b shows the associated CT spectrogram. In this experiment, the length of sensing path PWAS A to PWAS S1 is known. Besides, the group velocity dispersion curves of guided wave modes could be obtained since the features of the specimen are given (i.e., density: 2780 kg/m^3^, Young’s modulus: 73.1 GPa, Poisson’s ratio: 0.33). As a result, the time of arrival could be calculated from the following relation,

(28) $$T_{toa}\left(f\right)=T_{tof}\left(f\right)+T_{ac}\left(f\right),$$

where *T_tof_* = *s*/*v_g_*(*f*) is the time of flight, *s* is the length of sensing path, and *v_g_*(*f*) is the group velocity of a particular guided wave mode, as a function of the frequency. *T_ac_* is the launch time, which depends on the excitation signals. For a linear chirp, *T_ac_* = |*f* − *f*_0_|*T*/*B*, *f*_0_ is the starting frequency, *T* is the time duration, and *B* is the chirp bandwidth. As a function of the frequency, the time of arrival of S0 mode could be mapped into the time–frequency domain. In Figure 5b, it can be seen that the *T_to__a_*(*f*) (the white line) matches the time–frequency energy peak of S0 mode component exactly. On this basis, the instantaneous frequencies (IF) could be obtained from the *T_to__a_* relation.

Once the IF has been estimated, VKF is applied to extract the S0 mode component. In this experiment, the instantaneous bandwidth for each component takes 7.5 kHz. Figure 5c shows the extracted S0 mode response from the raw guided wave signal. Subsequently, the Fourier transform is employed to map the S0 mode response into the frequency domain. The transfer function of the guided wave inspection system is estimated as the ratio between that output and the linear chirp in frequency domain, and the amplitude dispersion curve could be obtained as the amplitude spectrum of the transfer function. Especially, the amplitude dispersion curve of S0 mode is shown in Figure 5d.

## 6. Results and Discussion

Due to amplitude dispersion, the performance of guided wave for damage detection would be affected. In this section, the influences of the amplitude dispersion to damage detection, and the effectiveness of the proposed compensation method are investigated.

### 6.1. Time Reversibility

In the time reversal method (TRM), an input signal is emitted from the source point, and then its output signal recorded at another point is reversed in the time domain and emitted back to the original source point [33]. The reconstructed signal *S*(*t*) could be calculated from Equation(9) as,

(29) $$\begin{array}{ll} s\left(t\right) & =\int_{-\infty }^{\infty }U^{*}\left(x,\omega \right)G\left(\omega \right)e^{j\left(\xi \left(\omega \right)x-j\omega t\right)}d\omega \\ & =\int_{-\infty }^{\infty }[F^{*}\left(\omega \right)G^{*}\left(\omega \right)e^{-j\xi \left(\omega \right)x}]G\left(\omega \right)e^{j\xi \left(\omega \right)x}e^{-j\omega t}d\omega \\ & =\int_{-\infty }^{\infty }{\left|G\left(\omega \right)\right|}^{2}F^{*}\left(\omega \right)e^{-j\omega t}d\omega \end{array},$$

where the superscript “*” denotes the complex conjugation operation.

In TRM, the detection of damage is achieved by measuring the discrepancy between the original input signal and this reconstructed signal [34,35,36]. However, due to the amplitude dispersion (i.e., *G*(*ω*) is a function of the frequency), the reconstructed signal would be different from the input signal even there is no damage in the wave path, and the time reversibility property may not be preserved.

In this section, the time reversal method is investigated by the transmitter–receiver pair in pitch–catch configuration (i.e., PWAS A to PWAS S2). Before the introduction of damage, a linear chirp with its frequency sweeps from 1 MHz down to 5 kHz over a rectangular window with the duration of 500 μs is applied as the excitation signal to obtain a broadband guided wave response. On this basis, the narrowband responses are extracted directly from the chirp response without multiple measurements, provided that their frequency bands belongs to the chirp bandwidth [25].

**Figure 6:**
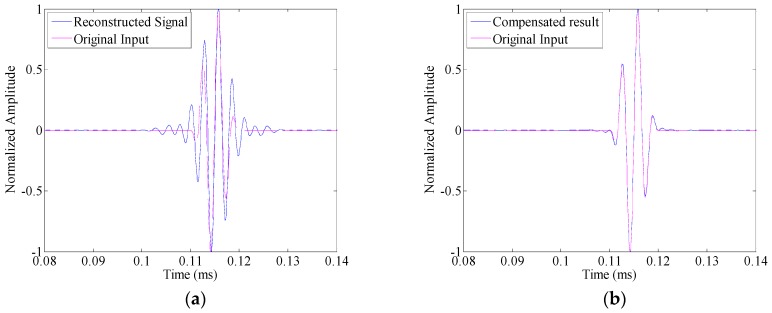
The comparison between the input signal and the reconstructed signal in the undamaged case: (**a**) without amplitude dispersion compensation; and (**b**) with amplitude compensation.

The time reversal process is investigated under narrowband excitations. Three-cycle toneburst signals with the center frequency changes from 250 kHz to 450 kHz with a step of 50 kHz are applied as the input signal, respectively. For illustration, Figure 6a shows the reconstructed signal as the center frequency of the toneburst signal takes 300 kHz. The input signal is also displayed in this figure for comparison. It can be seen that even in the undamaged case, the reconstructed signal is quite different from the input signal. Especially, the duration of the reconstructed signal is much larger than that of the input signal. It means that due to the amplitude dispersion, the time reversibility breaks down. Subsequently, the proposed compensation method (Section 4.2) is applied to the reconstructed signal. In metallic plate, since the material damping is not serious, the amplitude dispersion is not sensitive to the propagation distance. Hence, the amplitude dispersion curve shown in Figure 5d is applied as $\tilde{G}\left(\omega \right)$, and the polynomial order *N* takes 60. It is noted that the amplitude dispersion influences the time reversal process twice, i.e., both the forward and backward propagations. Hence, the variation *y* in Equations (24) and (25) should be $\tilde{G}{\left(\omega \right)}^{2}$. The comparison between the compensated result and the input signal is given in Figure 6b. It can be seen that with the amplitude dispersion compensation, the result matches the input signal quite well in the undamaged case. Hence, the time reversibility is preserved.

**Figure 7:**
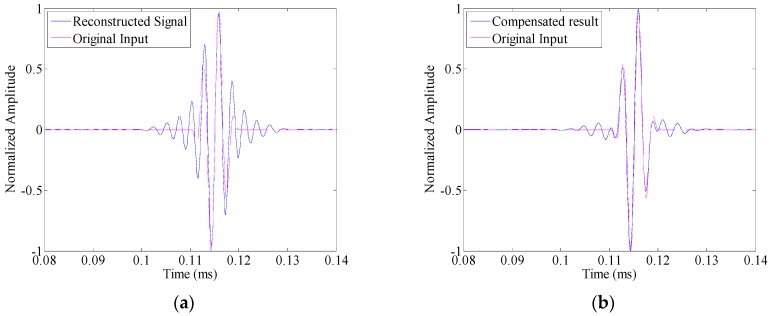
The comparison between the input signal and the reconstructed signal in the damaged case: (**a**) without amplitude dispersion compensation; and (**b**) with amplitude compensation.

In the damaged case (with the simulated crack marked as D in Figure 4), the same linear chirp excitation is also applied, so that the toneburst responses could be extracted from its response. Figure 7a shows the comparison between the reconstructed signal and the input signal. The differences between these two waveforms could be obviously observed, and they arise from both the damage and the amplitude dispersion. After amplitude dispersion compensation, the result is shown in Figure 7b. It is still different from the input signal. However, this difference is only attributed to the presence of damage.

Subsequently, a damage index (DI) based on the root mean square deviation is used to measure the relative distortion energy of the reconstructed signal*s* (*t*) compared to the original input *f*(*t*),

(30) $$\mathrm{DI}=\sqrt{{\left\{\int_{t_{0}}^{t_{1}}\left[s\left(t\right)-f\left(t\right)\right]dt\right\}}^{2}/\int_{t_{0}}^{t_{1}}s{\left(t\right)}^{2}dt},$$

where *t*_0_ and *t*_1_ define the signal length used for comparison. In this experiment, the time range [*t*_0_, *t*_1_] takes [0.08, 0.14] ms.

**Figure 8:**
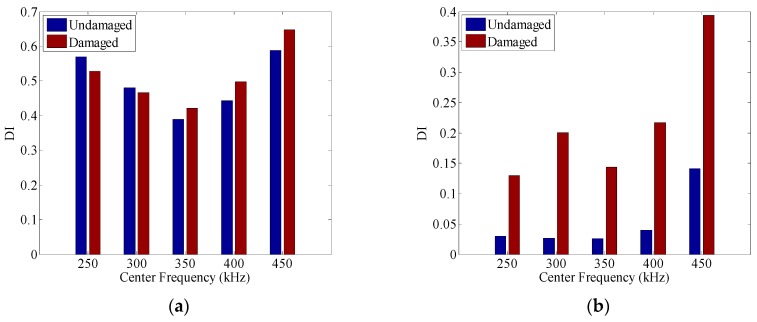
The comparison of the damage index between the undamaged case and the damaged case: (**a**) without amplitude dispersion compensation; and (**b**) with amplitude compensation.

Figure 8a shows the comparison of the damage index between the undamaged case and the damaged case without amplitude dispersion compensation. It can be seen that when the center frequency of the toneburst signals takes 250 kHz and 300 kHz, the values of damage index in the damaged case are even smaller than those in the undamaged case. It means that even though both the damage and the amplitude dispersion influence the waveform of the final signal and further the damage index, their interaction may not always lead to larger values of damage index. As a result, the TRM cannot identify the presence of damage at these excitations. In contrast, with the application of the amplitude dispersion compensation, the value of damage index at any excitation in the damaged case is larger than that in the undamaged case. The values of the damage index in both the undamaged case and the damaged case are listed in Table 1. It can be observed that after amplitude dispersion compensation, the sensitivity of the damage index to the damage (i.e., the increase of values of damage index from the undamaged case to the damaged case) is also enhanced. Hence, it can be concluded that the amplitude dispersion compensation could preserve the time reversibility of the TRM, and ensure its applicability for damage detection.

### 6.2. Testing Resolution

Referring to [37], the duration of a wave-packet and hence the resolvable distance of guided wave modes are governed by two terms, i.e., the increase in wave-packet duration due to dispersion *T_disp_* and the length of the input signal *T_in_*. To discuss the effects of amplitude dispersion to the testing resolution, Equation (9) is replaced by,

(31) $$u\left(x,t\right)=\int_{-\infty }^{\infty }\left[F\left(\omega \right)G\left(\omega \right)\right]e^{j\xi \left(\omega \right)x}e^{-j\omega t}d\omega =\int_{-\infty }^{\infty }\tilde{F}\left(\omega \right)e^{j\xi \left(\omega \right)x}e^{-j\omega t}d\omega ,$$

Here, $\tilde{F}\left(\omega \right)=F\left(\omega \right)G\left(\omega \right)$ is the new excitation signal. It means that due to the effects of amplitude dispersion, the duration of a wave-packet is the sum of *T_disp_* and the length of the new excitation signal *T_in_*’. As mentioned, due to amplitude dispersion, the shape of the new excitation signal would be different from that of the input signal, and thus the time duration varies.

**Figure 9:**
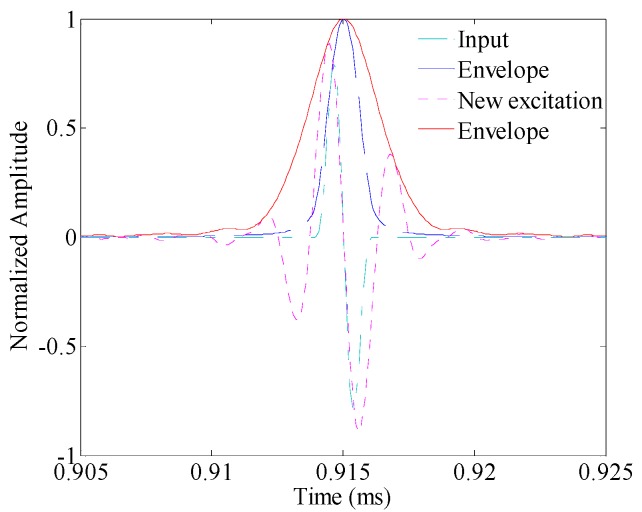
Comparison between the input signal and the new excitation signal for the two-cycle 450 kHz toneburst excitation.

In this section, the testing resolution is investigated by the transmitter–receiver pair in pulse-echo configuration (i.e., PWAS A to PWAS S1). The toneburst signals centered at 450 kHz but the cycle numbers 3, 2 and 1 are applied as the input signal, respectively. For instance, Figure 9 shows 2-count toneburst signal centered at 450 kHz. The associated new excitation signal is then calculated, and the result is also shown in Figure 9. It can be seen that the time duration of the new excitation signal is much larger than that of the input signal. Especially, the increase of cycle number could be obviously observed.

**Figure 10:**
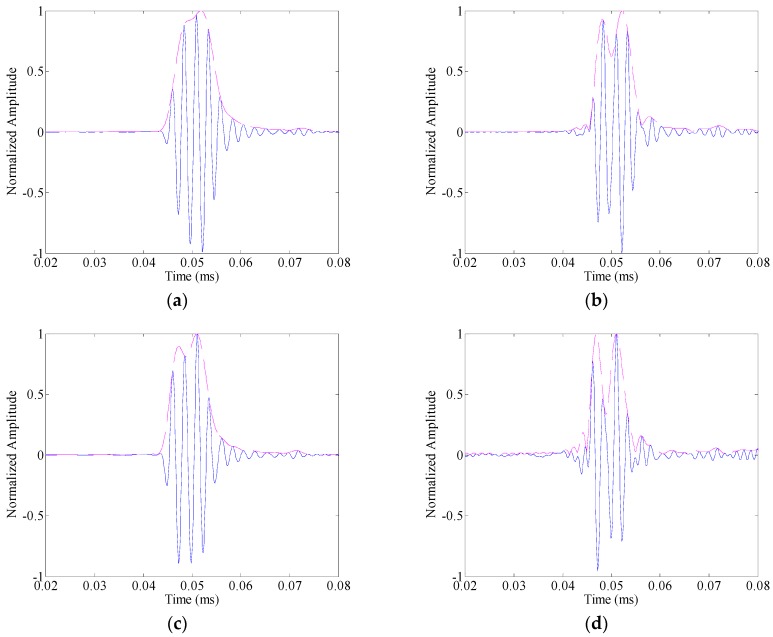

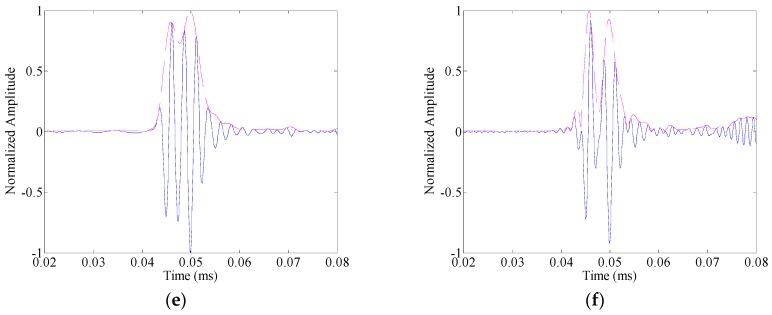
The guided wave responses (on the left hand side) and the associated compensated results (on the right hand side) under different toneburst excitations: (**a**,**b**) three-cycle toneburst; (**c**,**d**) two-cycle toneburst; and (**e**,**f**) one-cycle toneburst.

Figure 10a,c,e shows the responses as the cycle number of the toneburst takes 3, 2 and 1, respectively. It can be seen that the incident wave-packet (from the source) and the one reflected from the crack overlap with each other, and the defect cannot be correctly identified. With the application of amplitude dispersion compensation, the associated results are shown in Figure 10b,d,f, respectively. The evolution of time durations of the two wave-packets could be obviously observed from these figures. When the cycle number of the toneburst is 3, the two wave-packets still overlap with each other after amplitude dispersion compensation. As the cycle number decreases to 2, the overlap between the two wave-packets gets much weaker. Especially, at the amplitude 0.5, the time durations of the two wave-packets are 2.4 μs and 3.2 μs, respectively. When the cycle number is 1, the two wave-packets could be separately identified, and the time durations of the two wave-packets are 2.0 μs and 2.5 μs, respectively. The reason behind these phenomena could be explained as follows.

As the cycle number of the toneburst decreases, its time duration decreases. However, its bandwidth increases at the same time. As a result, both the effects of the velocity dispersion and amplitude dispersion enhance. In this case, the time duration of the received wave-packet may not decrease, and the testing resolution is limited. At the excitation frequency (i.e., [45, 855] kHz when the cycle number is 1), the group velocity of S0 mode only changes from 5431 m/s to 5244 m/s. Thus, the velocity dispersion term *Tdisp* does not change much with the bandwidth variation. In contrast, with the application of the amplitude dispersion compensation, the length of the new excitation signal *T_in_'* decreases obviously, and thus the time duration of the received wave-packet decreases and the testing resolution improves.

## 7. Conclusions

In this study, the effects of the amplitude dispersion are investigated, and a strategy is established to remove these effects and improve the performance of guided wave methods for damage detection. Some conclusions can be drawn from this work:

(i) The amplitude dispersion changes the spectrum of guided wave response, and influences the waveform of a propagating wave-packet.
(ii) With the application of the amplitude dispersion compensation, the time reversibility is preserved. On this basis, the damage could be correctly identified by the time reversal method.
(iii) The amplitude dispersion compensation method could compress the guided wave signals in time domain. Benefiting from that, the structural features located in the close proximity could be separately identified.

## Figures and Tables

**Table 1:** Damage index calculated in the undamaged case and damaged case.

Center Frequency	Without Amplitude Dispersion Compensation			With Amplitude Dispersion Compensation		
	Undamaged	Damaged	Increase of DI	Undamaged	Damaged	Increase of DI
250 kHz	0.5693	0.5280	−0.0413	0.0300	0.1295	0.0995
300 kHz	0.4804	0.4660	−0.0144	0.0265	0.2003	0.1738
350 kHz	0.3897	0.4210	0.0313	0.0260	0.1439	0.1179
400 kHz	0.4432	0.4974	0.0542	0.0400	0.2167	0.1767
450 kHz	0.5878	0.6477	0.0599	0.1412	0.3941	0.2529
